# Contact Transfer Printing of Side Edge Prefunctionalized Nanoplasmonic Arrays for Flexible microRNA Biosensor

**DOI:** 10.1002/advs.201500121

**Published:** 2015-06-24

**Authors:** Jihye Lee, Jiyun Park, Jun‐Young Lee, Jong‐Souk Yeo

**Affiliations:** ^1^ School of Integrated TechnologyYonsei UniversityIncheon406‐840South Korea; ^2^ Yonsei Institute of Convergence TechnologyYonsei UniversityIncheon406‐840South Korea

**Keywords:** contact transfer printing, hetero assembly, microRNA‐21, nanoplasmonics, side edge prefunctionalization

## Abstract

For a nanoplasmonic approach of wearable biochip platform, understanding correlation between near‐field enhancement on nanostructures and sensing capability is a crucial step to improve the sensitivity in biosensing. A novel and effective method is demonstrated to increase sensitivity with the enhanced electric fields and to reduce noise with targeted functionalization enabled by transferring side edge prefunctionalized (SEPF) nanostructure arrays onto flexible substrates. Nanostructure sidewalls have selective biochemically functional terminals for the hybridization of microRNAs (miRNAs) and the immobilization of resonant nanoparticles, thus forming hetero assemblies of the nanostructure and the nanoparticles. The unique configuration has shown ultrasensitive biosensing of miRNA‐21 in a 10 × 10^−15^ m level by a red‐shift in scattering spectra induced by a plasmon coupling. This ultrasensitive SEPF nanostructure arrays are fabricated on a flexible substrate using a contact transfer printing with a release layer of trichloro(1H, 1H, 2H, 2H‐perfluorooctyl)silane. The introduction of the release layer at a prefunctionalizing step has proven to provide selective functionalization only on the sidewalls of the nanostructures. This reduces a background noise caused by the scattering from nonspecifically bound nanoparticles on the substrate, thus enabling reliable and precise detection.

## 1 Introduction

Metallic nanostructures have gained great attention as noble metals strongly interact with an incident light leading to collective coherent oscillations of conduction electrons within nanoscale geometry, called localized surface plasmon resonance (LSPR).^1^, ^2^, ^3^ Such interaction results in an enhancement of electromagnetic field as well as maximum scattering at a resonant wavelength for a metallic nanostructure, which can be explained by a radiative theory.^4^, ^5^ The integration of nanoplasmonics has been demonstrated recently with stretchable, flexible, or biocompatible substrates in order to enable new functionalities for low cost, disposable, and wearable biosensors.^6^, ^7^ Achieving these features requires the engineered localization of electromagnetic fields so that the nanoplasmonic biosensing can provide reliable, sensitive, and selective detections^8^ for such examples as binding events of molecules,^9^, ^10^, ^11^ chirality of proteins,^12^ and precipitation of immunoassay‐based reactions.^13^, ^14^, ^15^

However, an adequately targeted functionalization to bind bioanalytes where the electromagnetic interactions are most intense and to suppress background scattering from polymer substrates is very important to improve the performance of the LSPR‐based flexible biosensor.^16^ Addressing the challenge will also bring the limits of detection to sub‐femtomolar concentrations.^17^ A nanostructure having a localized distribution of electric field, or a hot spot, can be targeted as a biorecognition site by hybridizing with nanoparticles to form hetero assemblies at a controlled interparticle gap.^18^, ^19^, ^20^ Specific and relevant examples are dimer,^21^ and core‐satellite structures^22^, ^23^, ^24^, ^25^ used for simple and robust colorimetric assay that can be explained with the generalized multiple Mie (GMM) solution.^26^, ^27^

In order to take advantage of these appealing approaches, we must be able to arrange the metallic hetero assemblies with consistent gap and to control their placement near hot spots on the flexible substrate by developing appropriate fabrication processes. Conventional approach is to use the chemically synthesized gold nanoparaticles (GNPs),^28^, ^29^, ^30^ and to immobilize GNPs on a transparent glass or polymer substrate using (3‐Aminopropyl)triethoxysilane(APTES)^31^ or organosilsesquioxane (GR720P in propoxypropanol).^32^ These widely accepted coupling agents require several conditions in order to place the nanoparticle assemblies effectively on a flexible and nonplanar substrate to achieve reliable plasmon coupling. A high quality monolayer silane is required to prevent random aggregation of the nanoparticles.^33^, ^34^ In addition, temperature and humidity levels should be finely controlled^35^ to keep the coupled nanoparticles in place after chemical and physical treatments.

Therefore, for the controlled fabrication of the nanostructure assemblies on a flexible substrate, it is desirable to provide an unconventional top‐down lithographic method beyond those bottom up approaches. Nanoimprint lithography^36^, ^37^ has been widely used for establishing a biosensing platform based on a quasi‐3D plasmonic crystal,^38^ and for controlling the sub‐10 nm spacing of nanostructure assemblies^39^ on a polymer substrate with high‐resolution.^40^ Nano stencil lithography (NSL),^41^, ^42^ namely, “resistless method,” has been proposed for the easy and intuitive way to transfer metallic nanodot arrays and nanowires onto a polymer substrate using a parallel shadow mask at low cost. Contact transfer printing^43^, ^44^ has been additionally demonstrated with highly uniform nanostructures on a flexible substrate for implantable or wearable biosensing applications.

However, these simple and robust top‐down fabrication approaches have encountered the following challenges. One is a direct control of the interparticle gap and the location among purposed particles.^39^, ^45^ The next challenge is an efficient post‐process^12^, ^46^ of keeping functional terminals on nanostructures for biorecognition while removing residual terminals from the polymer substrate.^8^, ^47^, ^48^ The residual terminals with nonspecified coupling result in an undesirable change of resonant wavelength. Another challenge is to realize nanoplasmonic sensors with appropriately engineered hot‐spots fabricated by scalable low cost processes. For a colorimetric sensing based on the core‐satellite assembly, conventional approach has been to reduce the size of core nanoparticles, thus requiring high resolution patterning on transparent and nonplanar substrates.^49^, ^50^ Smaller patterns of nanostructures generally lead to higher cost and lower yield in manufacturing processes. Alternative approach of utilizing a field enhancement from the side‐edge of nanostructures can ease the patterning requirement by controlling a thickness of the layer forming nanostructures rather than a width. Finally, a capability to selectively position the assemblies near the engineered hot‐spot is yet to be demonstrated to achieve maximum near‐field and detection sensitivity of biomolecules.^51^

In order to address these challenges, we present a new approach of fabricating an ultrasensitive nanoplasmonic sensor on a flexible substrate by contact transfer printing of side edge prefunctionalized (SEPF) nanostructure arrays. The strategy of utilizing the side edge of nanostructures for plasmon resonance allows the fabrication and replication in relatively larger nanostructure mold, thus enabling adequately scalable and reliable process. The approach of the side edge based field enhancement enables not only robust nano transfer printing but also efficient and selective binding of analytes around the sidewalls of the nanostructure arrays. Overall, this new approach can provide targeted functionalization on the purposed site to enable controlled assemblies with highly enhanced signal while reducing background noise by the carefully designed printing process flow utilizing the release of the metal nanostructures onto the flexible substrate with the tricholoro(1H, 1H, 2H, 2H‐perfluorooctyl)silane (FOTS) layer. The functionalized sidewall having thickness less than a plasmon length^52^ is where the electric fields are localized and enhanced as seen by finite‐element electrodynamic simulations. With the unique approach of prefunctionalization, nonspecific bindings of nanoparticles and biomolecules are effectively suppressed as observed in scattering images from dark‐field optical microscopy. We employ microRNA‐21 (miRNA‐21) as a target molecule since its over expression is an indicator of various cancers.^53^ There are a great number of excellent fabrication methods for miRNA biosensors with high sensitivity. The micro‐ring resonator,^54^ the nanowire‐based,^55^, ^56^ the nanopore‐based,^57^, ^58^ and the polymer‐based barcode assay^59^ have already been demonstrated. This nanotechnology‐based miRNA sensors show atto‐ or zepto‐mole level detection providing great sensing potential for cancer patients. However, they require complicated fabrication process and high density probe attachment. Our plasmonic sensing platform offers an adequate detection capability with a simple and low‐cost nanofabrication process for SEPF nanostructure on flexible substrate. The engineered SEPF nanostructure provides near‐field enhancement at the presence of hetero assemblies linked by miRNA‐21, resulting in the improved sensing performance of more reliable and accurate biomolecule detection for patients adjusting for clinical application.

## 2 Results and Discussion

### 2.1 Fabrication of SEPF Nanostructure Arrays and Hetero Assemblies

**Scheme 1:**
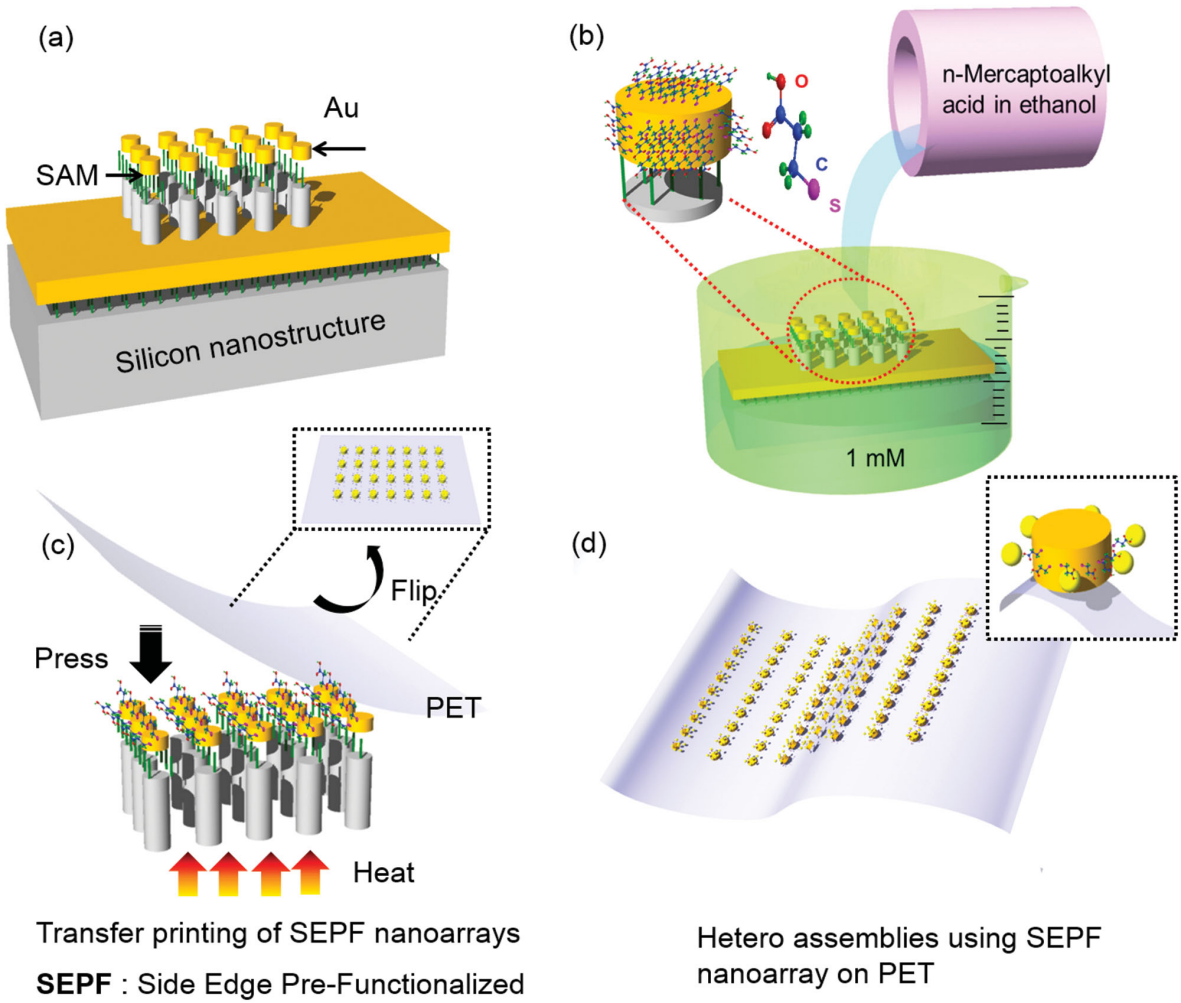
Schematic illustration of fabrication processes for the side edge prefunctionalized (SEPF) nanostructure arrays and the assemblies of heterostructures: a) coating of self‐assembled monolayer and 100 nm gold on silicon master, b) immersion of silicon master in *n*‐mercaptoalkyl acid ethanolic solution to form bioactive functional terminals on top and sidewalls, c) contact and release of the flexible substrate and the master on a hot plate resulting in carboxylic acid terminals exposed only on the sidewalls, d) attachment of satellite nanoparticles on the side edge prefunctionalized nanostructure arrays.

In the field of multifunctional LSPR biosensors, targeted functionalization on nanostructure and minimal background scattering are essential to obtain enhanced sensitivity and limit of detection (LOD). In order to achieve these properties, we exploited the SEPF nanostructure arrays and their fabrication processes using a relatively simple contact transfer printing method as shown in **Scheme** **1**. A silicon master or a Si mold with nanoscale patterns (as presented in the Experimental Section) was prepared and a self‐assembled monolayer (SAM) of gas phase tricholoro(1H, 1H, 2H, 2H‐perfluorooctyl)silane (FOTS) was coated on the pattern as a metal release layer.^60^, ^61^ After forming a SAM, polycrystalline gold (100 nm) was deposited by thermal evaporation (Scheme 1a). Subsequently, carboxylic acid terminal groups were activated by immersing the prepared master in a 1 × 10^−3^ m *n*‐mercaptoalkyl acid ethanolic solution for 12 h and heating it at a mild temperature of 50 °C (Scheme 1b). Various alkyl solution lengths were used such as 3‐Mercaptopropionicacid 99% (3‐MPA), 11‐Mercaptoundecanoic acid 98% (11‐MUA), and 16‐Mercaptohexadecanoic acid 90% (16‐MHA), all of which are well known to produce a stable organic surface on metallic films.^62^, ^63^ Mild heat (50 °C) was needed to prevent the dimerization of carboxylic acid from lowering the functional efficiency.^64^, ^65^ These conditions modify the top and side edges of the gold nanostructures chemically with terminal groups such as 5′‐thiolated miRNA probes and cationic ions to immobilize bio and chemical molecules with high affinity.

As a next step, the chemically treated master and a flexible substrate of 128 μm thick polyethylene terephthalate (PET) were placed on a hot plate at the temperatures of 80 °C (30 min) and 100 °C (2 h) under a pressure for the direct transfer of highly ordered SEPF nanostructure arrays (Scheme 1c). At this temperature range, the stability of the functional groups could be maintained.^66^ Then, the SEPF nanostructure arrays were finally fabricated on PET substrate with the functional terminals exposed only from the nanostructure sidewalls for the selective probing of target bioanalytes. Assemblies of the hetero structures were formed by linking nanoparticles with the SEPF nanostructures using either cationic Cu^2+^ ions or miRNAs. The Cu^2+^ ions and carboxylic acid group can be linked by electrostatic force (Scheme 1d). With plasmonic coupling effects arising from such assemblies, we have confirmed an ultrasensitive biosensing of miRNA‐21^35^ as will be explained in the Experimental Section in detail.

### 2.2 Analysis of Antiadhesion Layer on Si Mold and Transfer Printed SEPF Nanostructures

**Figure 1:**
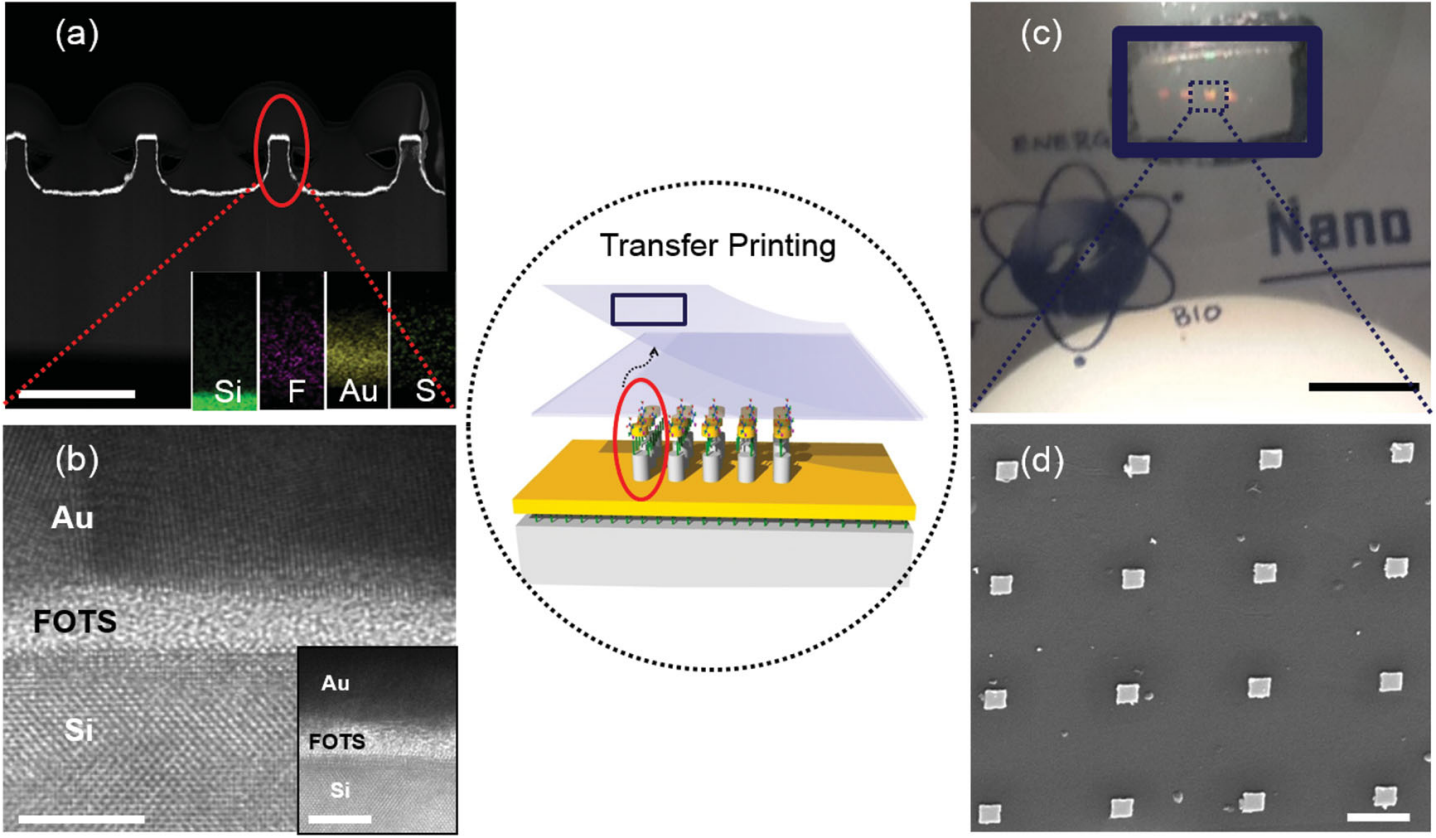
Analysis of the Si master prepared for the contact transfer printing of SEPF nanostructure arrays using a) cross‐sectional transmission electron microscopic (TEM) image of the silicon master (inset shows energy dispersive spectroscopic (EDS) results) (scale bar: 2 μm), b) high resolution (HR) TEM images after functionalization prior to the transfer (prefunctionalization) (scale bar: 5 nm) and HRTEM image (inset) of the Si master before prefunctionalization indicating no notable changes for the layers (scale bar: 5 nm). Analysis of the SEPF nanostructure arrays using c) photographic image of the transferred SEPF nanostructure arrays showing a scattering in red (scale bar: 5 mm, on a transparent PET substrate with a group logo on the background), d) scanning electron microscopic (SEM) image of the SEPF nanostructure arrays (scale bar: 1 μm)

Nanostructured Si master and layers on top were analyzed for their robustness, stability, and mechanical integrity after going through the processes with heat and functionalization in the contact transfer printing. The rigid master has a FOTS SAM and metallic gold layer as verified by transmission electron microscopic (TEM) images and energy dispersive spectroscopic (EDS) analysis (**Figure** **1**a). The FOTS, a prominent antiadhesion layer grows as a monolayer in vapor phase with the known properties such as length, roughness, and surface energy.^61^, ^67^, ^68^ The application of FOTS layer to contact transfer printing requires further study on its stability throughout the subsequent functionalizing processes such as chemically reactive solution and physical changes of temperature and pressure. High resolution TEM image in Figure 1b inset shows silicon, FOTS, and gold layer with their respective (011) single crystalline, amorphous, and polycrystalline phases before functionalization. The FOTS is either a mono­layer or a bilayer in approximately 2–3 nm long chain linked with silicon when the master is used for the first time. The length of the FOTS is changed to 7–8 nm after the repeated use of the master for 3 or 4 times (in igure S1, Supporting Information). In other words, the strong bond of Si–O–Si between silicon and FOTS is stable so that the layer remains even with the repeated exposure to O_2_ plasma at the mold cleaning step. When the mold is reused, application of each SAM coating leads to a vertically stacked structure of the amorphous polymer layers. This stacked structure induced by the bonding between alkyl chain of the FOTS and terminal fluorine chain is stable and does not deform during the contact transfer printing process so that the mold can be reused. Our results have verified the crystallinity of each layer and the stability of FOTS covalent bonds with the functionalization step, thus the potential to reuse the Si master repeatedly for transfer printing.

Figure 1b shows the functionalized master after immersion step in mercaptoalkyl acid solution. The lengths of 3‐MPA, 11‐MUA, 16‐MHA used for functionalization were about 7.82, 20.14, and 27.84 Å, respectively, as calculated by their molecular lengths and the angle of the atoms. The functional chains used in our experiments were not able to penetrate the densely packed FOTS monolayer so the chemically active chain was only attached to the side and top of the nanostructure retaining the robust FOTS property before the transfer. The gold layer was able to preserve its original polycrystalline state even with the functionalization process and 50 °C heat treatment, as verified by the TEM. This is crucial for the plasmonic application of the gold nanostructure after the transfer onto a flexible substrate. The stability of FOTS polymer film plays an important role not only as an antiadhesive layer for reliable transfer printing process but also as an intermediary layer to enable targeted functionalization on the sidewalls of metallic nanostructures if the three layers of FOTS, Au, and functional terminals are placed in an appropriate order on Si mold for the final transfer to the flexible substrate. Figure 1c–d shows optical and scanning electron microscopic images of the transferred SEPF nanostructure arrays on a flexible, transparent, and cost effective PET substrate. Figure 1c shows a photograph of the PET substrate illuminated with a fiber coupled high intensity halogen lamp (12 V/50 W). This figure shows the four patterns of the SEPF nanostructure arrays in the characteristic red color within the blue lined rectangle (each pattern contains 10000 arrays in 300 × 300 μm). The color of the sensing area on the PET indicates the spectra of plasmon resonance corresponding to the thickness of the transferred gold nanostructures (≈100 nm). The spectra from scattered photons are determined by the oscillation length of the conduction electrons so it is possible to control a resonance wavelength in the visible range by changing the width or the thickness of nanostructures for colorimetric biosensor.^8^ In our experiment, main resonance occurs along the side edge of the nanostructure, not the width. Along the width direction, a plasmon resonance cannot be induced due to the large dimension of the top surface compared to the plasmon length^52^ which is the total length of the collective electron oscillations in a given field. Scanning electron microscopic (SEM) analysis of the chemically modified nanostructure arrays on the PET is shown in Figure 1d. The size and pitch of the transferred gold nanostructures are defined by the dimension of the Si mold whose thickness and width are 100 ± 5 and 550 ± 50 nm, respectively.

### 2.3 Enhanced Field Distribution at SEPF Nanostructure

**Figure 2:**
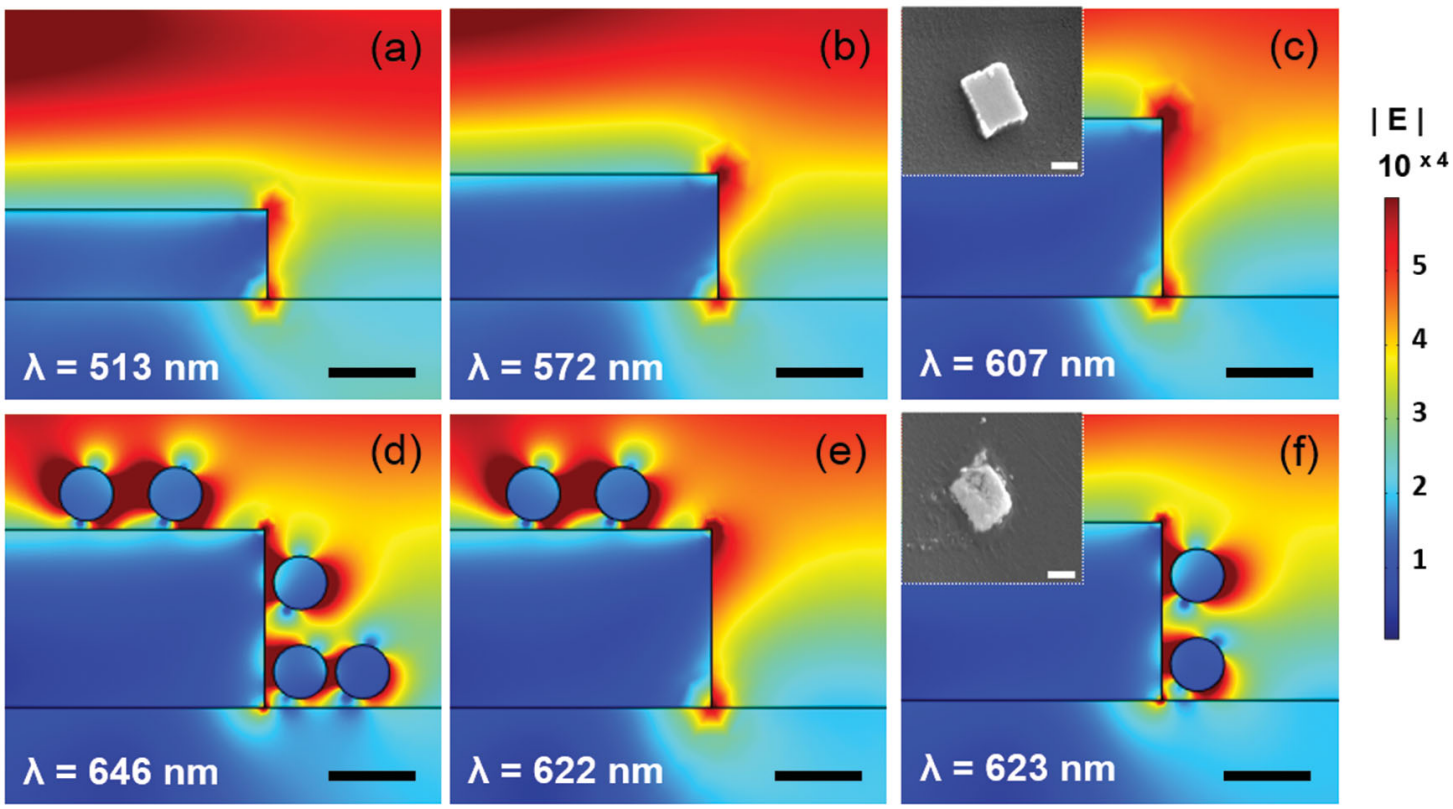
Distribution of the enhanced electric field amplitude at SEPF nanostructure on PET substrate using a finite‐element electrodynamic simulation for nanostructures with different thicknesses of a) 50 nm, b) 70 nm, and c) 100 nm, each showing maximum field distributions at the wavelengths of 513 nm, 572 nm, and 607 nm, respectively. Inset (c) shows SEM image of a single nanostructure (scale bar: 500 nm). Simulation of hetero assemblies of d) all the sides, e) the top, and f) the SEPF nanostructure with 30 nm satellite nanoparticles showing plasmonic coupling effect at the wavelength of 646, 622, and 623 nm, respectively (scale bar: 500 nm).

The thickness of the nanostructure crucially affects the distribution of the enhanced electric field along the sidewalls. As explained earlier, the main plasmon resonance occurs along the side edge having the thickness of approximately 100 nm. To investigate the region with the localized electric field, we have simulated the electric field distribution for various thicknesses and conditions with the SEPF nanostructures. **Figure** **2**a–c shows the nanostructures having thicknesses of 50, 70, and 100 nm, respectively. The near‐field enhancement is shown at the edge, corner, and sidewalls of the metal nanostructure at the resonance excitation wavelengths of 513, 572, and 607 nm for the increasing thicknesses of 50, 70, and 100 nm, respectively. In these simulations, field enhancement occurs along the sidewalls, especially on the edges called hot spots. Figure 2c inset shows the SEM images of the nanostructure on PET substrate with a 100 nm thickness having an extremely strong localized field distribution along the side edge. Since it is important to form the hetero assemblies on the edges where the field is enhanced, the shape, size, and number of edges as well as the spacing between them need to be optimized in order to improve sensitivity and limit of detection. A mold design with square patterned arrays of sub‐micrometer nanostructures has been adopted to facilitate printing and release process for efficient transfer and to provide effectively large surface area of sidewalls for forming hetero assemblies. Also, arrays with sufficiently large pitch are used to minimize cross‐talk among the plasmonic nanostructures.

As shown in Figure 2d–f, the 30 nm satellite nanoparticles attached with a 7 nm gap either on all the sides (Figure 2d), on the top only (Figure 2e), or on the prefunctionalized side edges (Figure 2f) of the nanostructures lead to the localization of the electric field in the coupled hetero assemblies. The 7 nm gap corresponds to the total length of the functionalization for the hetero assemblies: a combination of RNA probe 1 and 2 from the nanostructures and the nanoparticles, respectively, and hybridizing target miRNA with their lengths calculated by considering each sequence of the RNA base. Strong electric field along the side and top is distributed as shown in Figure 2d indicating a scattering wavelength of 646 nm. However, this localized field also stems from the coupling of nonspecifically bound nanoparticles randomly placed on the PET substrate. Figure 2e shows a weak field distribution at the wavelength of 622 nm from the dimer coupling between the nanoparticles attached on the top surface, not from the coupling between the nanostructure and the particles as the top surface is not where the plasmon is active.

On the other hand, the strong field localization from the hetero assemblies results in the resonance wavelength of 623 nm as shown in Figure 2f and the inset shows the scanning electron microscopic image of the hetero assemblies attached specifically along the side edges which were previously functionalized by the mercaptoalkyl acid. Therefore, targeted functionalization around the side edge of the nanostructures could be used as a simple and effective method to form a flexible nanoplasmonic platform for biosensing applications by incorporating the edge specific coupling effect.

Our SEPF nanostructure‐particle coupling shares a close similarity with a particle‐on‐film system.^69^ However, the particle‐on‐film system is caused by the coupling of surface plasmon polariton (SPP) and LSPR while our system is supported by the coupling of two LSPR resonants. For the particle‐on‐film system, there are two plasmon modes such as a tightly confined gap mode and a dipolar plasmon mode. They are determined by the characteristics of interaction depending on the presence of a gap. The excitation of plasmon mode is affected by the polarization of the illuminated light. As the nanostructure‐particle coupling also provides a gap, our geometry can be analyzed by the gap plasmon mode where the electric field is strongly localized and confined as shown in the simulated near‐field distribution in Figure 2f. The gap plasmon mode from the coupling of SEPF nanostructure and satellite nanoparticle can be maximized by illuminating light with its polarization parallel to the hetero assembly, which also results in a radiation with a faster damping rate.^69^

XPS data shown in Figure S2, Supporting Information, explains the binding energies of the functional layers and their stabilities compared between the conditions of the pre‐ and postfunctionalization.

### 2.4 Optical Responses of Background Noise from Substrate

**Figure 3:**
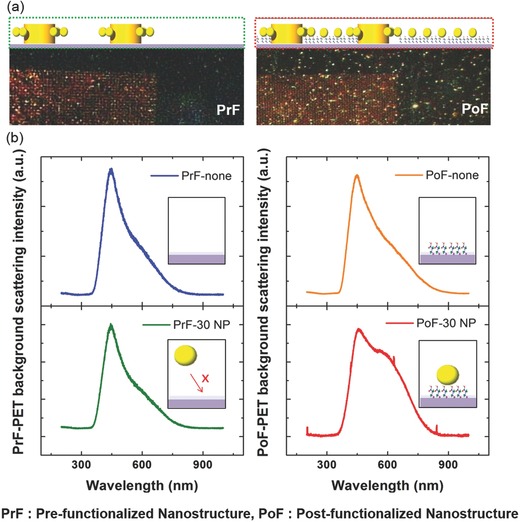
a) Dark‐field microscopic images of prefunctionalized (PrF) nanostructure arrays (left) and postfunctionalized (PoF) nanostructure arrays (right) on PET substrates. Prefunctionalization approach eliminates nonspecific binding of nanoparticles (dark‐region), thus suppressing background noise from the substrate. b) Spectrometric responses showing the PET background intensity and peak shift from the PrF (left) and PoF (right) nanostructure arrays before and after the application of 30 nm nanoparticles indicate nonspecific bindings of the nanoparticles leading to background scattering noise from the substrate when postfunctionalized, while the prefunctionalization and transfer of the nanostructures keep the background free of scattering on the PET substrate. (All the insets schematically represent the conditions associated with each spectral response.)

The targeted functionalization on the side edge of the SEPF nanostructures not only achieved the highest field confinement in the hetero assemblies for sensitive detection but also eliminated the negative influence of nonspecific binding that induces an irrelevant signal enhancement. These results allow us to accurately measure the interparticle gap associated with the near‐field coupling between the nanostructures and the nanoparticles.^47^, ^70^, ^71^ The benefits of the prefunctionalization can be seen by comparing the shift of peak wavelength and enhanced intensity at plasmon coupling between the two different conditions of functionalization for the metal nanostructures on the PET substrate. The samples are prepared for postfunctionalized (PoF) nanostructures using a conventional approach with chemically modified nanostructures and for prefunctionalized (PrF) nanostructures using our SEPF arrays which eliminate nonspecific binding on the polymer substrate. Fabrication of the PrF nanostructures follows the procedure described in Scheme 1. PoF nanostructures are fabricated by fully immersing the transferred nanostructure arrays on PET in a mercaptoalkyl acid solution. Dark‐field microscopic images show that there are major differences between the PrF and PoF nanostructures as shown in **Figure** **3**a. These images display that the hetero assemblies of 30 nm satellite nanoparticles and SEPF nanostructures are formed well by linking 3‐MPA molecular monolayers with cationic copper ions (Figure 3a, left). (The 3‐MPA is used since the spectral shift is largest for the shortest interparticle gap which is a function of alkyl chain length as shown in Figure S3, Supporting Information).

However, the image from the PoF nanostructures (Figure 3a, right) shows noise scatterings from the PET background as well as from the space separated by a microscale pitch between the nanostructures. The image of the noise scatterings from the dark region of the PET indicates the presence of the nonspecifically bound nanoparticles and the corresponding plasmon coupling. Further understanding of the background noise requires more detailed analysis of the scattering from a bare PET as shown in Figure 3b. Highly transparent polymeric PET shows strong scattering response at the peak wavelength of 450 nm.^72^ The left plot in Figure 3b shows that the scattered intensity and peak wavelength remain the same before and after applying 30 nm nanoparticles for the prefunctionalized (PrF) condition. In contrast, there is a distinctive difference in the scattered intensity and wavelength measured on PET before and after the treatment with nanoparticles for postfunctionalized (PoF) condition as shown in the right plot of Figure 3b. According to the data sheet from Sigma‐Aldrich, the peak LSPR wavelength of a single 30 nm nanoparticle is 526 nm while our measurement resulted in the wavelength of 546 nm. The shift of the single particle resonance was affected by the large refractive index of PET polymer substrate (*n* = 1.57).^41^ The background scattering from the nonspecific binding of nanoparticles hinders the efficient background correction and disturbs the gap‐controlled near field coupling with target nanostructures. In this regard, sensitive and accurate near‐field coupling can be made possible with prefunctionalization (PrF) using our SEPF nanostructures which will not require further data correction for the false peak shift. (See the Experimental Section for making hetero assemblies with the SEPF nanostructures.)

### 2.5 Optical Responses of Hetero Assemblies on Sensing Array

**Figure 4:**
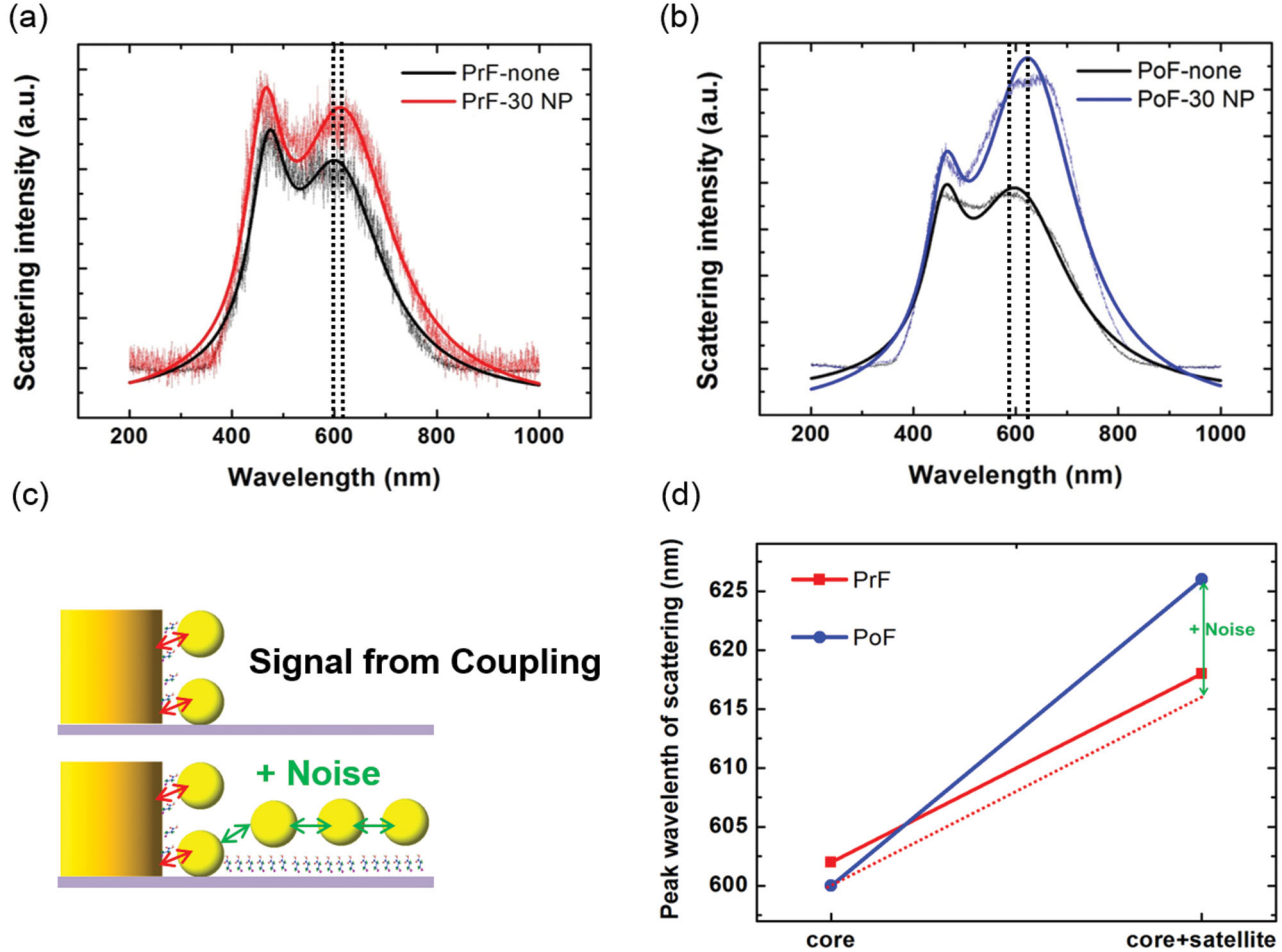
Scattering peak wavelength and intensity compared between prefunctionalized (PrF) and postfunctionalized (PoF) conditions. a) The red line shows the peak wavelength in scattering from the hetero assemblies composed of the PrF nanostructures and 30 nm satellite particles indicating a 16 nm spectral shift compared to black spectra from single nanostructure. b) The blue line shows the peak wavelength in scattering from the hetero assemblies composed of the PoF nanostructures and 30 nm satellite particles with a 26 nm spectral shift compared to black spectra from single nanostructure. c) Images illustrating the coupling schemes for the signal and the noise that result in the different shift of peak wavelength as shown in plot (d). d) Shift of peak wavelength in sensing region where the 30 nm nanoparticles are attached to the PrF and PoF nanostructures, respectively. The peak shift of 10 nm is a noise induced by undesirable plasmon coupling among the satellite nanoparticles attached on background PET substrate.

The results of the shift in peak wavelength from the scattering spectra illustrate that the nonspecific binding of nanoparticles strongly influences the plasmon resonance as shown in **Figure** **4**. Scattering intensity and peak shift are quite different between the PrF in Figure 4a and PoF in Figure 4b nanostructures. Peak wavelength induced from the PrF sensing region is changed from 602 to 618 nm with a 16 nm peak shift depending on the number of attached nanoparticles, whereas the PoF sensing region shows a large change in resonant wavelength from 600 to 626 nm as well as larger increase in intensity. As illustrated in Figure 4c, this large increase is due to the noise coupling with additional nanoparticles attached on the postfunctionalized substrate. These observed red‐shifts scattering from the coupling can be understood from generalized GMM theory.^26^, ^27^ The shift from the coupling between the SEPF core and 30 nm satellite particles is expected to increase linearly as more satellite particles are attached.^73^ However, the shift of 10 nm shown in Figure 4 is too large to be explained by the linear increase (Figure 4d). The dark‐field scattering image from the PET background in Figure 3a on the right indicates that the larger shift should be a noise induced by undesired coupling among the satellite nanoparticles attached on the substrate. The coupling among the satellites can also contribute to the larger increase in intensity. The intensity and peak shift corresponding to the number of coupled satellites require more complicated analysis and deconvolution of the data for correction.^22^ Therefore, it is desirable to eliminate the source of noise from the sensing platform itself, which again validates the approach of the transfer printed SEPF nanostructures.

### 2.6 Ultrasensitive miRNA‐21 Detection Using Hetero Assemblies on Uniform Substrate

**Scheme 2:**
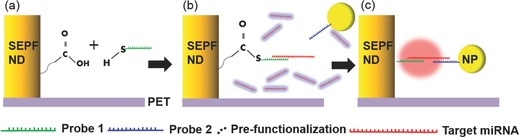
Biofunctionalization on the SEPF nanostructures conjugated with 3‐MPA, probe 1, target miRNA, and probe 2.

To investigate the biosensing capability using this SEPF nanostructure, sample was prepared with noncoding miRNA‐21 which is a promising noninvasive biomarker for the pathogenesis of various diseases found in body fluids.^74^, ^75^, ^76^ Two RNA probes were prepared and attached to the nanostructures and satellite nanoparticles, respectively, for sensing miRNA‐21. Modified SEPF nanostructures were conjugated by RNA probe 1 and satellite GNPs were linked by RNA probe 2. (Detailed procedures for the conjugation of RNAs are described in the Experimental Section shown in **Scheme** **2**. Additionally, information on the strand of RNA base is also shown in **Table** **1**.) These two resonant structures and target miRNA can be hybridized by two probing strands when the amount of miRNA concentration is sufficient to form hetero assemblies. In other words, the arrays of SEPF nanostructures having the width of 500 nm and the height of 100 nm were used for selectively identifying the miRNA‐21 in femtomolar to picomolar concentration with plasmon coupling. The gap distances between the SEPF ND and 10, 30 nm sized NPs were 7–8 nm for the hybridizing experiment with miRNA‐21. (Detailed information is proved in the Experimental Section.)

**Table 1:** Target miRNA‐21 and strand information of two oligonucleotides

Length	Symbol	Sequences
22‐mer	Target miR‐21	5′‐UAGCUUAUCAGACUGAUGUUGA‐3′
12‐mer	Probe 1	5′‐Thiol‐UUUUCAACAUCA‐3′
12‐mer	Probe 2	5′‐GAUAAGCUAUUU‐Thiol‐3′

**Figure 5:**
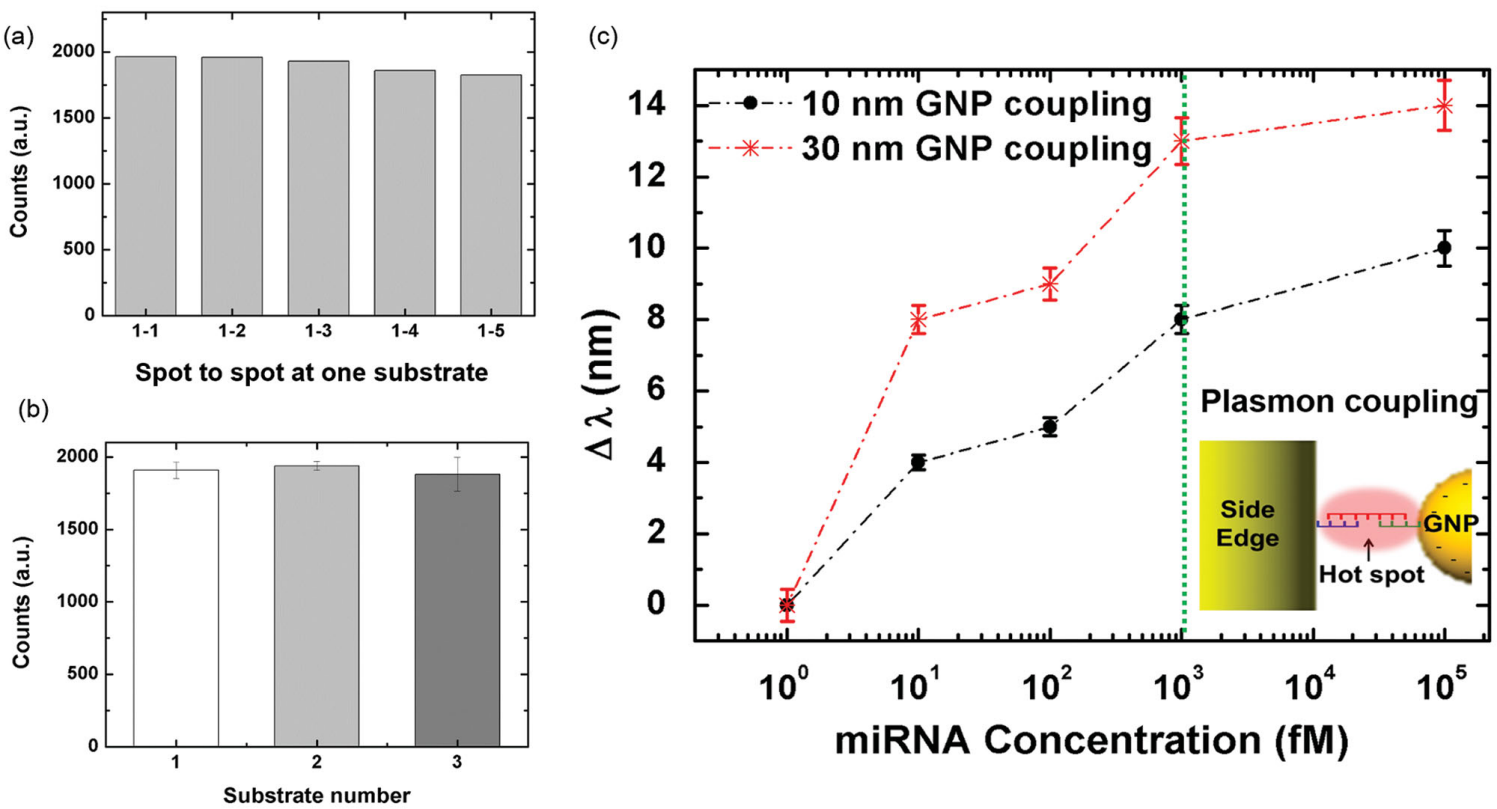
Examination of reproducibility and uniformity for spot‐to‐spot and substrate‐to‐substrate conditions using SEPF nanostructures and its application for miRNA detection. a) A plot of spot‐to‐spot variations in a sample showing uniformity. b) A plot of substrate‐to‐substrate variations from three samples showing reproducibility. c) Shift of scattering spectra response to the miRNA concentration ranging from 1 × 10^–15^ m to 100 × 10^–12^ m. Spectral shifts show consistent difference between 10 and 30 nm satellite particles due to their relative sizes for plasmon coupling. (Inset: A schematic of plasmon coupling with the SEPF nanostructure and the satellite GNPs linked by target miRNA‐21 using two probes of complementary strands.) The dotted green line indicates the early detection level of miRNA for general patients.

Uniformity and reproducibility of the fabricated substrate are evaluated by measuring average signal intensities with spot‐to‐spot and substrate‐to‐substrate variations. We have calculated the average intensity and coefficient of variation (CV) to plot the data as shown in Figure S4, Supporting Information. All the scattering intensities are measured at 602 nm wavelength. After three measurements in five spots from one sample, the average intensity is 1908.7 counts with a 2.9% CV as shown in **Figure** **5**a. This histogram shows that the spot‐to‐spot variations indicate the highly uniform intensity distribution among the randomly selected spots from a substrate. In Figure 5b, three flexible substrates each with 250000 SEPF nanostructures have been measured for substrate‐to‐substrate signal variations. The 1940.0 counts with 1.6% is measured from substrate 2 and 1880.6 counts with 6.2% is from substrate 3. Compared to other results from a literature,^77^ our substrates have shown smaller values of CV ranging from 6.2 to 1.6. The small CV values in scattering intensity indicate that our SEPF nanostructures on a flexible substrate provide not only high reproducibility in nanofabrication but also uniformity within a fabricated substrate for miRNA biosensors.

Specificity of our biosensor toward microRNA‐21 detection has been addressed already in our previous work on the core‐satellite approach.^35^ Results from a number of clinically relevant microRNAs, but different from our target miR‐21, such as miR‐16, miR‐122, miR‐126, miR‐141, and miR‐206 have been presented by other researchers.^78^ Additionally, miR‐15 and miR‐16 are structurally similar to our target miRNA‐21 in the 5′region.^55^, ^79^ While these miRNAs have a possibility of attaching on probe 1, they are not capable of hybridizing to the probe 1 and 2 simultaneously, thus proving the specificity of our core‐satellite system for miRNA‐21 detection.

Figure 5c shows a plot for the shift of peak wavelength versus the miRNA‐21 concentration ranging from 1 × 10^–15^ m to 100 × 10^–12^ m with a detection limit of 10 × 10^–15^ m. Peak wavelength of 600 nm resulted from SEPF nanostructure was red‐shifted to 610 and 614 nm using a 10 and 30 nm conjugated GNPs, respectively, at the target miRNA‐21 concentration of 100 × 10^–12^ m. The 30 nm satellite GNPs resulted in a larger shift of peak wavelength due to the larger plasmon resonance length. Minimum detectable concentration was 10 × 10^–15^ m with the shift of wavelengths in 4 and 8 nm for 10 and 30 nm nanoparticles, respectively. According to the plot in Figure 5c, sufficient concentration of miRNA from 10 × 10^–15^ m leads to the formation of hetero assemblies contributing to the spectral shift in the given geometry of the nanostructure arrays. The trend in the plotted graph shows a nonlinear relationship between the concentration of miRNA‐21 and the shift in scattering wavelength. When the concentration is very low as in the case of 10 × 10^–15^ m miRNA, the shift of wavelength is determined by the size of the satellite nanoparticles (10 and 30 nm) attached to the SEPF nanostructures. As the concentration increases from 10 × 10^–15^ m to 1 × 10^–12^ m, more nanoparticles are attached to the available probes on the side of the nanostructure, thus increasing the scattering wavelength according to the number of satellites. For the range of concentration between 1 × 10^–12^ and 100 × 10^–12^ m, the scattering wavelength is starting to shift less as the finite surface area and probes become limitations compared to the larger number of nanoparticles with target miRNA‐21. The larger satellites of 30 nm are showing more saturating behavior than the smaller ones of 10 nm since the larger satellites not only provide larger size dependent shift for each nanoparticle but also consume the available surface area faster with increasing concentration.

Based on our results, the dynamic range in a practical diagnosis can be further increased by fabricating nanostructures that provide larger surface area for higher density of probes and more side edge with hot spots, and by selecting nanoparticles of optimal size that provide both sufficient wavelength shift in lower concentration and less saturating behavior in higher concentration.

For clinically relevant applications, it is important to understand how the sensitivity of our biosensor would change as we use biological samples. Although biological samples such as cancer cell line, human blood, urea, and serum with abundant protein (albumin, immune protein, glycoprotein, etc.) can cause the nonspecific bindings that affect the change of scattering wavelength especially in optical‐based sensors, previous results^55^, ^78^, ^80^ suggest that the wavelength shift with respect to the concentration of miRNAs from a serum shows a similar trend with the ones from RNA free water or TE buffer, showing a slight difference in the shift. Based on this trend, it is known that a 1 × 10^–12^ m level^78^, ^81^ (the dotted green line in Figure 5c) is sufficiently meaningful for early detection in clinical application. Since our SEPF ND based biosensing platform demonstrated the 10 × 10^–15^ m level of detection with a noise free background, we believe this can also be applied to the detection of miRNA‐21 in other physiological media.

In summary, miRNA sensing platform for a wide range of concentration using the hetero assemblies resulted in a sensitive, selective, and reliable detection with a limit of 10 × 10^–15^ m range comparable to practical bio sample concentrations.

## 3 Conclusion

For novel flexible nanoplasmonic sensors, we have demonstrated an ultrasensitive biosensing platform exploiting the transfer printing of SEPF nanostructures on a flexible PET substrate. This new approach consists of three major components with advantages. First, we have fabricated prefunctionalized nanostructures with biochemically active functional terminals for the hybridization of miRNAs and immobilization of resonant nanoparticles, thus forming hetero assemblies. Second, the nanostructure provides enhanced electric field on side walls so the side edge is selectively functionalized and then transferred by printing onto a flexible substrate in order to enhance a signal while reducing a noise. Finally, this unique configuration has shown ultrasensitive biosensing for a 10 × 10^−15^ m miRNA‐21 based on a red‐shift in scattering spectra from the plasmon coupling in the hetero assemblies with noise free detection. By optimizing the geometry of the nanostructure and the distribution of the arrays such as thinner sidewall and more edges for field enhancement, larger sidewall area, and optimal density of nanostructures for more binding sites and minimal cross‐talk between the adjacent hetero assemblies, our flexible biosensing platform is expected to allow further enhancement of the sensitivity. The unique SEPF nanostructure arrays also can be extended to various applications such as sensitive strain gauge sensor on the flexible substrate,^82^ functional molecular devices,^83^ functionalized particle based photoconductance,^84^ and more sensitive and selective biomolecule sensing interfaces integrated on diverse flexible substrates with localized surface plasmon resonance.

## 4 Experimental Section

*Materials*: To fabricate the SEPF nanostructure arrays, 3‐Mercaptopropionicacid 99% (3‐MPA), 11‐Mercaptoundecanoic acid 98% (11‐MUA), 16‐Mercaptohexadecanoic acid 90% (16‐MHA) and tricholoro(1H, 1H, 2H, 2H‐perfluorooctyl)silane (FOTS) were purchased from Sigma‐Aldrich. Ethanol (99.5%) was purchased from J. T. Baker Solutions. Flexible substrate, polyethylene terephthalate (PET), was purchased by Graphene platform (188 μm thickness, 92% light transmission with 0.9% haze, coefficient of thermal expansion MD 0.9%, TD 0.7%). To immobilize the hetero nanoparticles on the SEPF nanostructure, copper(II) sulfate solution of 0.1 m (Sigma‐Aldrich) and 10, 30, 150 nm nanoparticles (BBI international) were prepared. In order to detect the miRNA, oligonucleotides (probe 1 and 2 for recognizing the miRNA) and miRNA‐21 were purchased from Bioneer Co., Ltd with purification. RNase‐free water was purchased from Invitrogen. Nap‐10 column (GE Healthcare) and dithiothreitol (DTT) (Thermo Scientific) was purchased for purification without dimerization of the thiolated oligonucleotide. Tris‐HCL (hydroxymethylaminomethane) buffer (pH 7.5) (Thermo Scientific) and NaCl (Sigma‐Aldrich) was used for stabilization of conjugated oligonucleotide with nanoparticles.

*Conjugation of Probes onto SEPF and Satellite Nanoparticles and Hybridization of Target miRNA‐21 onto two Probes*: Hetero assemblies are made by hybridization of target miRNA at two RNA probes with matching complementary strands. The information on the strand of target miRNA‐21 and the oligonucleotides of two probes are shown in Table 1. Chemical reactions between carboxylic acid terminals and thiolated 5′RNA were utilized to facilitate these assemblies. The thiolated RNA strands were easily tangled with other thiols making a disulfide group. So we used the DTT which plays a role in preventing single sulfide from forming disulfide group. Scheme 2a shows consecutive reactions between RNA strand (Probe 1) and carboxylic acid. (Our probe concentration was used at 10 × 10^−6^ m (10 nm in 1 mL) for highly packed functionalization.) The main route of this reaction is condensation of thiol and carboxylic acid in the presence of dehydrating agents such as *N*,*N*′‐dicyclohexylcarbodiimide (DCC). But RNA free water also plays a role in dehydrating a carboxylic acid that controls the pH state. Therefore, the reactions occur for making a thioester within 1 h. After this reaction, we have prepared the probe 2 linked with nanoparticles as shown in Scheme 2b. Conjugation of probe 2 and citrate capped nanoparticles was done with additional stabilizing solution of 1 mL Tris‐HCL (hydroxymethylaminomethane) buffer (pH 7.5) (0.01 m) and NaOH (0.05 m). Conjugation of oligonucleotide with nanoparticles took about an hour to conjugate with thiolated RNA and nanoparticles. The following procedure is a preparation of target miRNA‐21 with a concentration gradient. We diluted the miRNA ranging from 1 × 10^–15^ m to 100 × 10^–12^ m to investigate the wide dynamic range of detection capability. The dilute water was RNA free water. Final procedure was the hybridization of target miRNA with two probes. Hybridization took about an hour to conjugate the target miRNA with probe 1 and took another hour to conjugate with the probe 2 on nanoparticles. The particles are connected with two probes and target miRNA‐21 composed of 3‐mercaptoalklyacid (7.82 Å), oligonucleotides (one base pair is approximatly 3.4 Å length, which corresponds to 643 daltons. Each base pair of two probes is 12. miRNA is composed of 22 pairs) in total length of 7–8 nm. Completed structure was linked as SEPF nanostructure‐Probe 1‐Target miRNA‐Probe 2 as shown in Scheme 2c.

*Fabrication of Nanoscaled Silicon Master*: The silicon master was prepared by the maskless photolithography (NanoSystem Solutions, Inc. D‐LIGHT DL‐1000) whose resolution limit is 1 μm. To achieve nano‐sized patterns in silicon using a micropatterned gold mask, reactive ion etching (RIE) was conducted significantly for etching (Source 200 W, Bias 50 W, O_2_ 20 sccm, SF_6_ 100 sccm, N_2_ 20 sccm,). In this process, the nanostructure was formed by controlling the power for the source and bias and by selecting proper mask materials. The 500 nm width and 650 nm height nanostructures with 3 μm pitch arrays were used as a final dimension for the silicon master.

*Simulation*: Simulations were carried out using a commercial software package (COMSOL Multiphysics, RF module) which solves the electromagnetic wave in a frequency domain. To investigate the electric field distribution on the fabricated nanostructure, we simplified the structures using the measured data from AFM. Nanostructure arrays were simulated as nano‐rectangles separated by a gap of 3 μm and satellite nanoparticles were simulated as nanocircles. The nanorectangles and the nanocircles were periodically repeated with microsized gap. These nanoscale objects were illuminated with a plane wave propagating along the *z*‐axis. Depending on the dimension and geometry of the objects, the mesh was optimized with the element size ranging from 33 to 0.14 nm.

*Characterization*: To characterize the silicon mold, transferred SEPF nanostructure on PET, and SAMs of FOTS, we have used Spherical aberration (Cs)‐corrected scanning transmission electron microscopy (Cs‐corrected STEM) and high resolution transmission electron microscopy (HRTEM) (JEOL, JEM‐ARM200F), field emission electron microscopy (FE‐SEM) (JEOL, JSM‐7100F), and atomic force microscopy (AFM) (Park Systems, XE‐100). Inverted optical microscope (Nikon, Ti‐U) with a dark‐field condenser (NA = 0.80–0.95, Dry type) and digital camera (Nikon, DS‐Ril) were used for dark‐field imaging to identify scattering of nanostructures on PET. A source of optical measurement is a halogen lamp with a neutral color balance filter. Spectra studies were carried out using a spectrophotometer (Dong Woo Optron Co., Ltd, Monora 320i) with a charge‐coupled device (CCD) (Andor Technology, DV420A‐BV) which was connected to the inverted optical microscope. The chemical analysis was carried out by X‐ray photoelectron spectroscopy (XPS) from Thermo Fisher Scientific (K‐ALPHA ESCA SYSTEM) with monochromatic Al K*_α_* X‐ray source. The analysis area was 400 × 400 μm^2^ on each sample.

## Supporting information

As a service to our authors and readers, this journal provides supporting information supplied by the authors. Such materials are peer reviewed and may be re‐organized for online delivery, but are not copy‐edited or typeset. Technical support issues arising from supporting information (other than missing files) should be addressed to the authors.

Supplementary
