# Supplementary material for: Contact Transfer Printing of Side Edge Prefunctionalized Nanoplasmonic Arrays for Flexible microRNA Biosensor
Source: Adv Sci (Weinh). 2015 Jun 24;2(9):1500121. doi: 10.1002/advs.201500121 (PMC5115393; doi:10.1002/advs.201500121)
Supplement: Supplementary file 1 — Supplementary [file ADVS-2-0m-s001.pdf]

Copyright WILEY-VCH Verlag GmbH & Co. KGaA, 69469 Weinheim, Germany, 2015.

ADVANCED SCIENCE
Open Access

Supporting Information

for *Adv. Sci.*, DOI: 10.1002/advs.201500121

Contact Transfer Printing of Side Edge Prefunctionalized Nanoplasmonic Arrays for Flexible microRNA Biosensor

*Jihye Lee, Jiyun Park, Jun-Young Lee, and Jong-Souk Yeo**

Supporting Information

Title Contact transfer printing of Side Edge Pre-Functionalized Nanoarrays for flexible nanoplasmonic miRNA biosensor

Jihye Lee[ab]*, Jiyun Park*[ab]*, Jun-Young Lee*[ab]*, and Jong-Souk Yeo*[ab*]

[a] School of Integrated Technology, Yonsei University, Incheon, 406-840, Rep. of Korea

[b] Yonsei Institute of Convergence Technology, Yonsei University, Incheon, 406-840, Rep. of Korea

E-mail: jongsoukyeo@yonsei.ac.kr

Keywords: nanoplasmonics; contact transfer printing; hetero assembly; side edge pre-functionalization; miRNA-21

Supporting Information 1

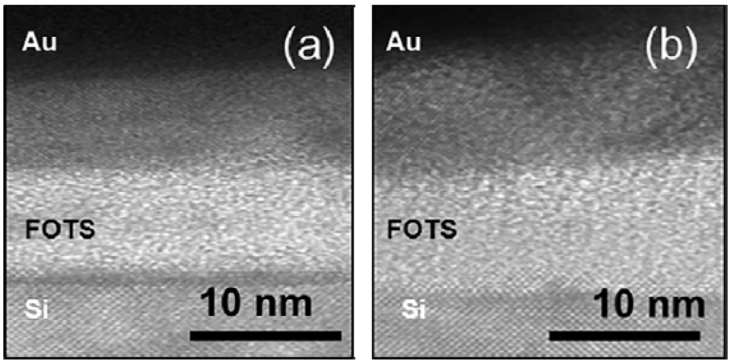

Figure S1. (a) High resolution transm ission electron m icrographs (HRTEM) of crystalline silicon, am orphous FOTS, and polycrystalline gold layer before functionalization and (b) HRTEM image of the Si m aster after pre-functi onalization using mercaptoalkyl acid solution. Lengths of the FOTS range from 7 to 10 nm after repeated use of the master for 3 to 4 times.

Supporting Information 2

Analysis of the side edge pre-functionalized (SEPF) nanostructure

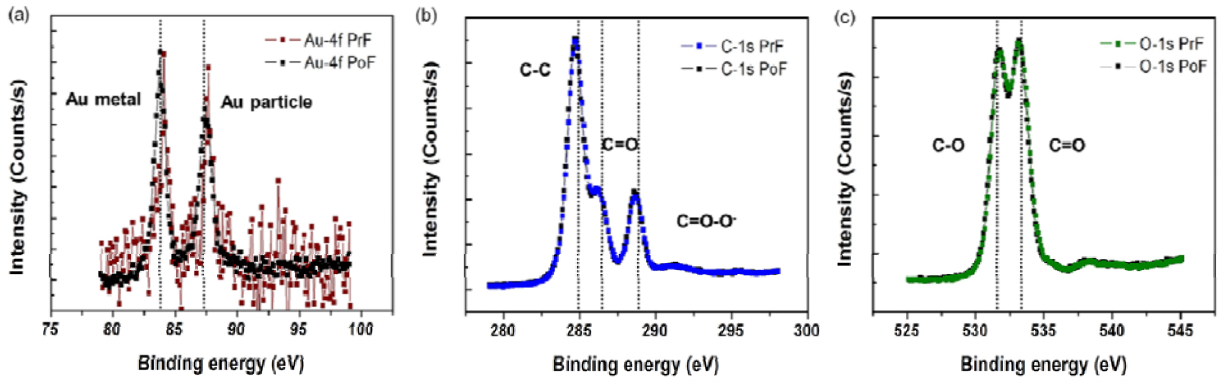

Figure S2. XPS data analysis compared between PrF and PoF conditions, (a) Au-4f spectra, (b) C-1s spectra, and (c) O-1s spectra of the SEPF nanostructures on flexible PET substrate.

The SEPF nanostructure was characterized by analyzing XPS data shown in Figure S2. Binding energies of Au metal and nanoparticles were shown at 84 eV and 86 eV, respectively (Figure S2 (a)). It is more important to check the attachment of functional molecules on the gold nanostructure whose role is to recognize a bioanalyte. C-1s spectra were ranging from 284.8 eV of C-C bond, 286 eV of carbonyl bond to 288.5 eV of anionic bond from purposed carboxylic acid as shown in Figure S2 (b). From this data, transferred gold nanostructures were confirmed to retain probing molecules on their sidewalls so that it enables a link with the cationic group or functionalized 5'-thiolated mi-RNA strands. This anionic terminal can also be utilized as a probing site to bind various analytes and can be modified further for bio-binding molecules. From XPS, O-1s spectra such as organic C-O and C=O bonds were placed at 531.5~532, 533 eV, respectively as shown in Figure S2 (c). The data indicates that there are no differences in the nanostructure properties between the pre-functionalization (PrF) and post-functionalization (PoF) so that the interaction between target biomolecules and binding site should not be affected. Therefore, the chemical analysis shown in Figure S2 (a)-(c)

confirms that the pre-functionalized gold nanostructure was well transferred onto a flexible PET substrate providing a high quality side-edge probing terminals.

Supporting Information 3

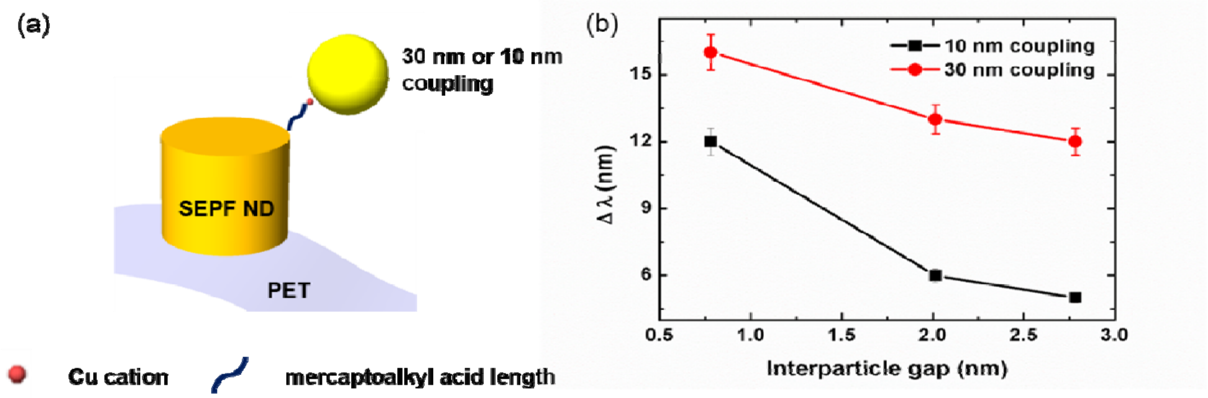

Figure S3 (a) Schematic shows hetero assembly of the SEPF nanostructure with the carboxylic acid terminals and the citrate-capped satellite nanoparticle (10, 30 nm) linked by copper cations using electrostatic interaction. (b) Shift of peak wavelength is inversely proportional to the interparticle gap mainly determined by the length of alkyl chain on the SEPF nanostructure terminated with carboxylic acid. The lengths of alkyl chains are 3-MPA (7.82 Å), 11-MUA (20.14 Å), and 16-MHA (27.84 Å), respectively. Shift of peak wavelength depends not only on the size of coupled nanostructure and nanoparticle but also on the interparticle gap between them. In this particular experiment, large-sized satellite particles with larger spectral shift were used to analyze the effect of various alkyl chain lengths on plasmon coupling. Since the shortest interparticle gap resulted in the largest shift in peak wavelength, we have selected the functionalizing condition with 3-MPA for the remaining experiments in the main paper.

Supporting Information 4

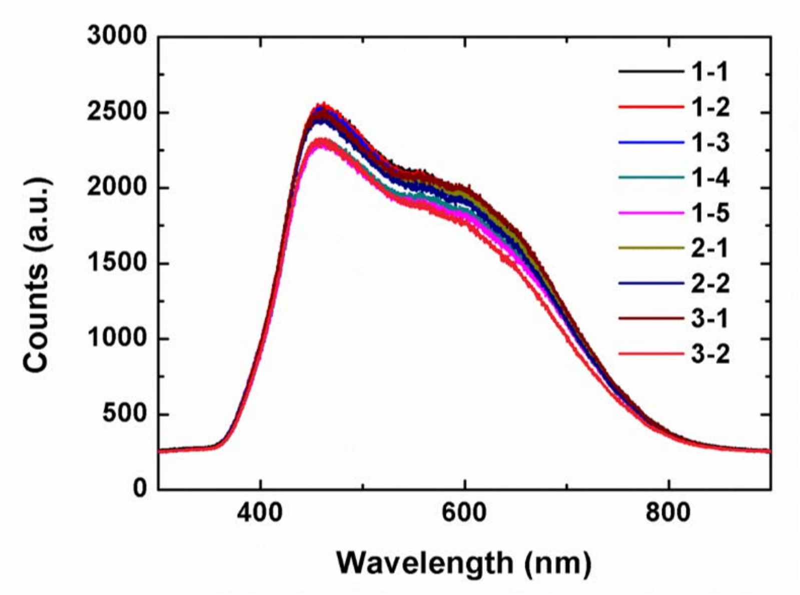

Figure S4. Examination of reproducibility and uniformity for spot-to-spot and substrate-to-substrate conditions. Scattering intensity is measured in the wavelength range from 300 to 900 nm for various spots on different substrates.
